# Electrogalvanism in Oral Implantology: A Systematic Review

**DOI:** 10.1155/2022/4575416

**Published:** 2022-08-05

**Authors:** Meriem Amine, Wiam Merdma, Khalid El Boussiri

**Affiliations:** ^1^Fixed Prosthodontics Department, Faculty of Dentistry of Casablanca, Hassan II University of Casablanca, Casablanca, Morocco; ^2^Faculty of Dentistry of Casablanca, Hassan II University of Casablanca, Casablanca, Morocco; ^3^Fundamental Biomaterials Department, Faculty of Dentistry of Casablanca, Hassan II University of Casablanca, Casablanca, Morocco

## Abstract

**Purpose:**

The objective of this work is to study galvanic corrosion of different couples of prosthetic and implant alloys through the realization of a systematic review.

**Materials and Methods:**

An electronic search was performed on Pubmed, Google Scholar, Scopus, ScienceDirect, EbscoHost, and Web of Science for published studies related to electrogalvanism in oral implantology. The keywords used were “dental implants” and “galvanic corrosion.” Two independent readers read the scientific articles.

**Results:**

From 65 articles initially identified, only 19 articles met the eligibility criteria. The evaluation of the selected articles allowed us to determine the parameters compared, such as the resistance to galvanic corrosion, the influence of fluorine and pH on the electrochemical behavior, and the release of metal ions and their cytotoxicity. Indeed, Ti6Al4V and precious alloys coupled to titanium were found to be the most resistant to galvanic corrosion, followed by cobalt-chromium alloys and nickel-chromium alloys which were least resistant. This resistance decreases with increasing fluorine concentration and with decreasing pH of the environment. *Discussion*. The implant-prosthetic system's galvanic resistance is influenced by many intrinsic factors: alloy composition and surface condition, as well as extrinsic factors such as pH variations and amount of fluorine. The effects of oral electrogalvanism are essentially the result of two main criteria: effects due to electric currents generated by corrosion and effects due to the release of metal ions by corrosion.

**Conclusion:**

To avoid this phenomenon, it is wise to follow the proposed recommendations such as the use of the minimum of distinct metals as much as possible, favoring the commercially pure titanium implant of Ti6Al4V, opting for the choice of couples, titanium/titanium, favoring daily mouthwashes of 227 ppm of fluoride, and avoiding fluorinated acid solutions.

## 1. Introduction

Electro galvanism is the result of the coupling of different metals or alloys with different corrosive potentials in an aqueous conducting environment (= electrolytic) [1, 2].

The sustainability of the implant-prosthetic complex depends on the osseointegration of the implant and the stability of the surrounding soft tissue. Morphology and surface roughness have a great influence on osseointegration.

The presence of microgaps within the system because of ionic release caused by galvanic corrosion can lead to the accumulation of bacterial biofilm on these surfaces. The dispersion in the tissue of particles of titanium oxide or other derivatives triggers an inflammatory reaction of the nonspecific immune system which certainly activates the resorption of the bone, causing long-term damage to the implant. The level of peri-implant inflammation affects the survival of the implants in the long term [3, 4].

The galvanic corrosion of biomaterials, used in oral implantology, in direct contact with the oral environment depends not only on their own properties but also on their interactions with their environment [5, 6].

In the light of the abovementioned facts, the objective of our work is to study the galvanic corrosion of different pairs of prosthetic and implant alloys, through the realization of a systematic review.

## 2. Materials and Methods

A literature search was conducted using the databases: Pubmed, Google Scholar, Cochrane Library, Science Direct, EbscoHost, Web of Science, Embase, and Clinical Trials. The keywords used were “dental implants” and “galvanic corrosion.” The search languages used were English and French. Any study dealing with the galvanic behavior of prosthetic alloys when coupled with implant alloys was selected. Studies dealing with galvanic couples in orthodontics and orthopedics were excluded. Studies dealing with other types of corrosion other than galvanic corrosion were also excluded. Two independent readers read the scientific articles.

## 3. Results

**Figure 1:**
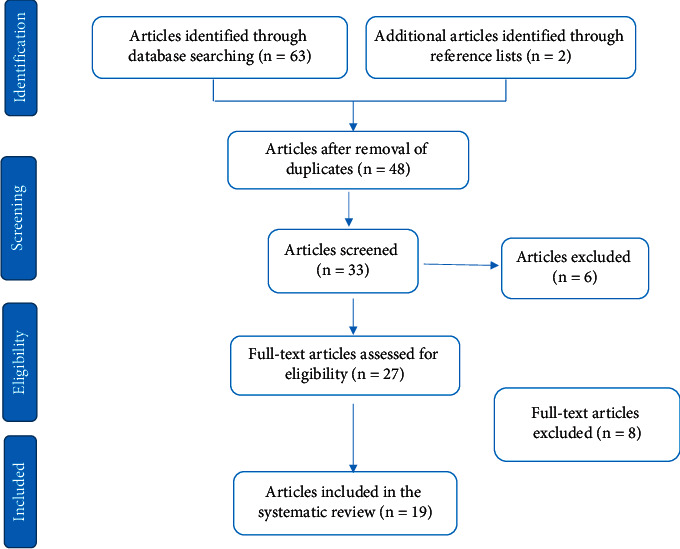
PRISMA flowchart for study selection.

The search of the scientific literature yielded 65 articles. We identified 19 studies that met our inclusion and exclusion criteria, of which only one was in vivo and the rest were in vitro (Figure 1).

The evaluation of the selected articles allowed to determine the parameters compared, namely the resistance to galvanic corrosion of the different couples (Table 1), the influence of fluorine and pH on the electrochemical behavior (Table 2), the oxidation surface state on titanium (Table 3), and the release of metal ions (Table 4) and their cytotoxicity.

### 3.1. Cytotoxicity

The decrease in the cell growth rate allowed Lee et al. to report that the cytotoxicity of nickel-chromium alloy with beryllium was greater than that of nickel-chromium alloy without beryllium. The addition of beryllium is therefore detrimental to the cellular activity of the tissues surrounding the implant. On the other hand, increasing the chromium content in the composition of the nonprecious nickel-chromium alloy has a beneficial effect on cytotoxicity [22].

## 4. Discussion

### 4.1. Factors Influencing Corrosion Phenomena

**Figure 2:**
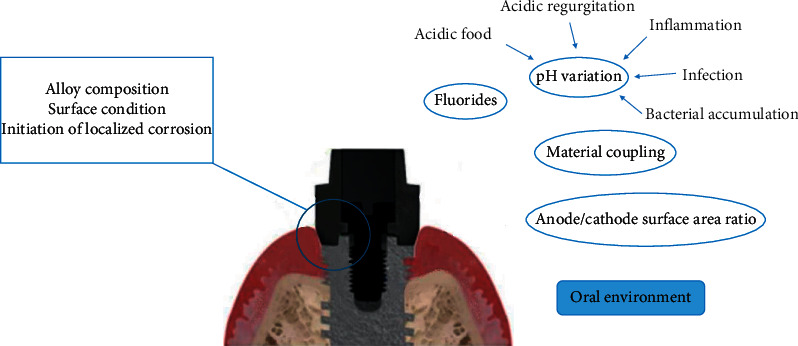
Intrinsic and extrinsic factors influencing corrosion phenomena.

The resistance of a metal or an alloy to corrosion depends not only on its own properties but also on its interactions with its environment. There are different factors influencing the corrosion of an alloy (Figure 2).

#### 4.1.1. Intrinsic Factors

*1. Alloy Composition*. Cp Ti implants have excellent biocompatibility and good galvanic corrosion resistance but low mechanical strength, whereas Ti6Al4V has high mechanical properties but low galvanic corrosion resistance [10, 11, 25].

Excellent galvanic corrosion resistance due to the high thermodynamic stability of gold characterizes high gold alloys.

The addition of palladium greatly improves the corrosion resistance of silver alloys. Alloys based on gold and palladium have a lower dissolution rate and therefore a higher corrosion resistance than those made of nonnoble base metals such as NiCr or CoCr [12–15, 18–20, 26].

*2. Surface Condition*. Oxide film thickness, energy, roughness, and grain size on the titanium surface influence corrosion resistance, biomaterial interaction with cells, and osseointegration mechanisms [7, 27, 28].

The acid-etch surface treatment of Ti cp directly affects the formation of galvanic couples, improves osseointegration, and increases corrosion resistance in the oral environment [7].

*3. Initiation of Localized Corrosion*. The initiation of other types of corrosion removes the passivation oxide layer and is likely to aggravate galvanic corrosion by increasing the current [17, 19].

#### 4.1.2. Extrinsic Factors

*1. pH Variations*. The pH of the environment plays a major role in the electrochemical behavior of the different couples; as the pH of the saliva decreases, the values of the galvanic current between the implant and its superstructure increase [2, 7, 21].

However, the normal pH of saliva secreted by the salivary glands varies between 6 and 7. It can reach acidic levels of about 2 when acidic foods are ingested or when acid regurgitation occurs, as it can vary in the areas around surgical sites and dental implants [29, 30].

*2. The Amount of Fluorine*. Prophylactic toothpastes, mouthwashes, and gels contain 200 to 20 000 ppm F− and may impair the corrosion resistance of prosthetic and implant dental alloys in the oral cavity [2, 5, 7].

In fact, increasing the concentration of fluoride ions decreases the corrosion resistance of titanium implants except at 227 ppm F− at pH 5.5, which is the fluoride concentration found in daily mouthwash [21].

It has also been shown that the combination of low pH and the presence of fluoride ions in the solution severely affects the degradation of the protective passivation layer that normally exists on titanium alloys, resulting in galvanic corrosion [7, 21, 29, 31].

*3. The Coupling between Implant Titanium and Prosthetic Superstructures*. When coupling, compatible metals should be selected for direct contact with each other in the oral cavity to avoid or minimize the formation of undesirable electrochemical couples [32].

The use of titanium alloy prosthetic superstructures on titanium implants avoids the problem of galvanic corrosion. A study by Arismendi et al. suggests that the best restoration-implant pairing can be achieved by using cp titanium and a titanium alloy [8]. Whereas Taher et al. suggest that the best couples are Ti/Ti, Ti/Or, and Ti/CoCr [15].

*4. Cathode/Anode Surface Area Ratio*. The most unfavorable situation is when a small anode is linked to a large cathode. This ratio can cause more corrosion [17].

### 4.2. Host Response to Electrogalvanism in Oral Implantology

The effects of oral electrogalvanism are primarily the result of two main factors:

- Effects due to electrical currents generated by corrosion
- Effects due to the release of metal ions by corrosion

#### 4.2.1. Osteolysis Induced by Electrical Currents Generated by Corrosion

It has been shown that cyclic loads (chewing and biting) enhance electrical currents induced by corrosive events. It is suggested that surrounding tissues are chronically exposed to abnormal electrical signals [33].

The bone responds to electrical potentials applied to it, and osteogenesis is proportional to electronegativity [34].

#### 4.2.2. Osteolysis Induced by Corrosion Debris

Olmedo et al. observed that corrosion-induced ion release may be responsible for periimplantitis and treatment failure [35].

Periimplantitis is characterized by a loss of the supporting bone, both clinically and radiologically proven, and is associated with an inflammatory reaction of the surrounding soft tissue [2, 36].

**Figure 3:**
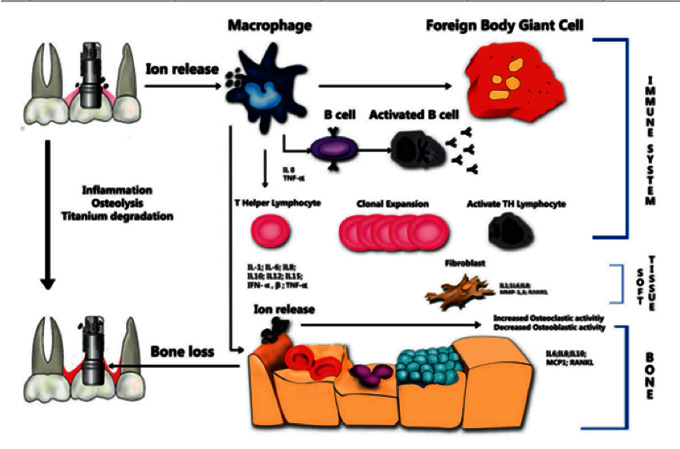
Process of ion release.

The metal ions released because of the corrosion process are phagocytized by macrophages and release inflammatory mediators in the form of pro-inflammatory cytokines, such as tumor necrosis factor alpha (TNF-*α*) and interleukin-1 (IL-1), and increased intercellular adhesion molecules (ICAM-1), which inhibit osteoblast production and promote osteolytic activity through the RANKRANK ligand pathway, thus inducing osteolysis of peri-implant tissues (Figure 3) [2, 31, 37–41].

Trace metals from implants have been shown to disrupt homeostasis (e.g., DNA synthesis, mineralization, and alkaline phosphatase mRNA expression). These traces have been found in the liver, lungs, lymph nodes, and bloodstream [33, 41–43].

## 5. Conclusion

There is a wide range of materials to be used in implantology, both for the implants and the superstructure, and the most effective treatment of electroplating in oral implantology remains preventive. The judicious choice of materials is made when establishing the prosthetic treatment plan.

The proposed recommendations to practitioners are as follows:

- The use of biocompatible materials and a minimum of discrete metals whenever possible
- The choice of metal couples whose elements are as close as possible in the galvanic scale; the best couples are Ti/Ti, Ti/Or, and Ti/CoCr
- The use of supra-implant ceramic restorations
- Prefer cp Ti to Ti6Al4V as an implant material for its better resistance to galvanic corrosion
- Avoid as much as possible, the direct contact between two different metals with cathodic inhibitors, a joint, an insulator, and a coating.
- Avoid an unfavorable anode-cathode surface ratio
- Avoid acidic fluoride solutions, especially when the implant is made of titanium alloy and the superstructure is made of Co-Cr, and therefore, prefer daily mouthwashes of 227 ppm fluoride

## Figures and Tables

**Table 1:** The evaluation of corrosion resistance of the different galvanic couples.

Study	Galvanic couples	Environment, pH, period, method, area ratio, etc.	Results
Soares et al. 2021 [7] in vitro	Annealed microstructured cp Ti G4/CoCrMo	(i) 0.9% NaCl and BB at 225 ppm F at pH 6 and 2	(i) Acid-treated cp Ti G4 and UFG Ti exhibited better corrosion resistance compared to cp Ti G4
	Cp Ti G4 acid treated/CoCrMo UFG Ti^1^/CoCrMo	(ii) Naturally airy.	(ii) The galvanic couple with the lowest current was nanostructured Ti cp in contact with CoCrMo alloy
		(iii) 24 H	
		(iv) OCP^2^ and ZRA^3^	
		(v) 0.2	
Mellado–Valero et al. 2018 [2] in vitro	Ti G2/Au	(i) AS^4^, SAF pH 6.5, and SAF pH 3	(i) The NiCrTi alloy shows a very narrow passive
	Ti G2/NiCrTi	(ii) OCP, CP^5^, and ZRA.	(ii) Domain, exhibiting transpassive dissolution at most
	Ti G2/CoCr	(iii) 30 min for OCP and hours 4 for CP	(iii) Low potential values compared to other materials
	Ti G2/CoCr-c	(iv) 0.28: CoCr-c and NiCrTi	(iv) The TiG2/Ti6Al4V couple shows no galvanic effect
	Ti G2/Ti6Al4V	(v) 0.5: CoCr, Ti6Al4, and AuPd	
Bortagaray et al. 2016 [8] in vitro	Ti cp/noble alloys	(i) AS pH 7, 1	Noble alloys with high gold and palladium content combined with cp titanium implants showed high resistance to galvanic corrosion
		(ii) Analytical technique by static immersion—3 months	
	Cp Ti/Cp Ti		
Ziębowicz, A. et al. 2015 [9] in vitro	Cp Ti c/Ti6Al4V	(i) A mandibular bone in	Galvanic corrosion hardly occurs in case of coupling between Cp Ti/Ti6Al4V
		(ii) Tyrode's solution, 37 ± 1°C	
		(iii) CP, and EIS^6^	
		(iv) 6 months	
		(v) 1	
Sola C. 2013 [10] in vitro, Anwar, E.M. et al. 2011 [11] in vitro	Cp Ti/noble alloys (Pontor®2)	(i) AS, pH of 7.1–37°C	The noble alloy/Ti couple proved to be the most resistant galvanic couple, whereas the noble alloy/Ti6Al4V couple presents the lowest corrosion resistance
	Ti 6Al4V/noble alloys (Pontor®2)	(ii) OCP, CP, EIS	
		(iii) 24 H	
		(iv) 0.9	
	Cp Ti/metallic ceramics (NiCr)	(i) SA pH of 7.5 -(ii) NaF added to AS: 3 different concentrations were tested	(i) The best corrosion resistance was presented by the cp Ti pairs compared to the other pairs where the implant was Ti6Al4V
	Cp Ti/ceramics		(ii) Titanium implants paired with ceramic-ceramic
	Ti 6Al4V/CM (NiCr)		(iii) Crowns showed the highest corrosion resistance rates compared to the other pairs tested
	Ti 6Al4V/ceramics	(iii) M NaF, M 0,05 NaF and M 0, 1 NaF	(iv) However, the best couple was cp Ti/ceramic
		(iv) OCP and EIS	
Tuna et al. 2009 [12] in vitro	Cp Ti (G4)/Pd	(i) AS, pH 6.7, at 37°C	The cp Ti G4/noble alloys pair showed a galvanic corrosion potential value significantly lower than that of the cp Ti G4/CoCr and cp Ti G4/NiCr pairs and therefore a better resistance to galvanic corrosion
	Cp Ti (G4)/Au	(ii) PD^7^, OCP,	
	Cp Ti (G4)/NiCr	(iii) 14 H	
	Cp Ti (G4)/CoCr	(iv) 0.33	
Arslan H. et al. 2008 [13] in vitro	Ti 6Al4V/Au	(i) Ringer at 37°C	The Ti6Al4V/Au pair had the highest resistance to galvanic corrosion, while the Ti6Al4V/NiCr couple presented the least
	Ti 6Al4V/NiCr	(ii) Absence of oxygen	
	Ti 6Al4V/CoCr	(iii) Cp, mixed potential theory	
Oh et Al 2004 [14] in vitro	Ti cp (G3)/Au	(i) AS at 37°C	The Ti cp (G 3)/Ti cp (G 3) and Ti cp (G3)/gold pairs exhibited relatively low passive current densities. While the implant pairs Co-Cr/Ti and NiCr/Ti had the highest values
	Ti cp (G3)/NiCr	(ii) OCP, PS^8^, and PD	
	Ti cp (G3)/CoCr	(iii) 5 000s	
	Ti cp (G3)/Ti cp (G3)		
Taher and Jabab 2003 [15] in vitro	Ti cp (G1)/Au	(i) AS fusayama modified at pH: 7,2	The best couples were Ti/Ti cp, Ti/Or and Ti/Co- Cr, while the Ti/Ni–Cr couple showed unstable galvanic corrosion behavior
	Ti cp (G1)/NiCr	(ii) Potentiostat	
	Ti cp (G1)/CoCr	(iii) 24 H	
	Ti cp (G1)/cp Ti (G1)	(iv) 0.78	
Cortada et al. 2000 [16] in vitro	Ti cp (G1)/Au	(i) AS, pH: 6.7 at 37°	The titanium implant coupled with a nickel-chromium alloy releases a large amount of ions and the implant coupled with the titanium superstructure has low values of released ions
	Ti cp (G1)/Pd	(ii) OCP, PD, potentiostat	
	Ti cp (G1)/NiCr	(iii) 250 min	
	Ti cp (G1)/Ti cp (G2) cast	(iv) 1	
	Ti cp (G1)/Ti cp (G2) machined		
Grosgogeat et al. 1999 [17] in vitro	Ti cp/CoCr	(i) AS aerated AFNOR pH at 6.737°C	(i) The most unfavorable situation is when a small anode is linked to a large cathode
	Ti 6Al4V/CoCr	(ii) AS deaerated fusayama 37°C, pH 5	(ii) There are other possible types of corrosion to consider in addition to galvanic corrosion, such as pitting and crevice corrosion
		(iii) OCP, PD, and potentiostat	
		(iv) 24 H for OCP	
		(v) 15 H for ZRA	
		(vi) 1	
Venugopalan and Lucas 1998 [18] in vitro	Cp Ti (G2)/Au	(i) AS fusayama 37*°*C, pH 5	(i) Precious alloys (based on au, Ag, and pd) coupled with titanium were found to be the least susceptible to galvanic corrosion
	Cp Ti (G2)/AgPd	(ii) OCP and PD	(ii) NiCr and CoCr based alloys coupled to titanium were moderately susceptible to galvanic corrosion
	Cp Ti (G2)/CoCrMo	(iii) 6 hours	(iii) Mo added to Ni–Cr based alloys plays a protective role against corrosion while Be has a negative influence
	Cp Ti (G2)/NiCrMo		
	Cp Ti (G2)/NiCr		
	Cp Ti (G2)/NiCrBe		
Reclaru and Meyer 1994 [19] in vitro	Cp Ti G4/Au	(i) AS fusayama pH 5, 37°C	(i) The coupling of titanium with nonprecious alloys presents a negligible risk with respect to crevice corrosion
	Cp Ti (G4)/CoCr	(ii) OCP and PD	(ii) Mo added to non-precious alloys plays a protective role against corrosion
	Cp Ti (G4)/FeNiCr	(iii) 24 H	
	Cp Ti (G4)/NiCrMo	(iv) 1	
Ravnholt 1988 [20] in vitro	Cp Ti/Au	(i) Solution de NaCl à 1% aérée	No corrosion current was recorded when gold and CoCr were in contact with titanium
	Cp Ti/CoCr	(ii) pH 6,25 ± 0.25 à 37 ± 1°c	The changes occurred when the amalgam was in contact with the titanium
		PD, potentiostat	
		20 days	

^1^UFG Ti: ultrafine grained titanium; cold worked nanostructured cp Ti G4; ^2^OCP: open circuit potential; ^3^ZRA: zero-resistance-ammeter; ^4^AS : artificial saliva; ^5^CP: potentiodynamic curves; ^6^EIS : electrochemical impedance spectroscopy; ^7^PD : potentiodynamic curves; ^8^PS : potentiostatic test.

**Table 2:** The influence of fluorinated and acidic media on the galvanic corrosion resistance of the different galvanic couples.

Study	Galvanic couples	Environment, pH, period, method, area ratio, etc.	Results
Soares et al. 2021 [7] in vitro	Microstructured annealed Ti G4/CoCrMo	0.9% NaCl and BB at 225 ppm of F^2^ at pH 6 and pH 2 Naturally airy. 24 HOCP^3^, ZRA^4^.0.2	(i) The corrosion resistance of the different couples decreased in the mouthwash solution.
	Acid-treated Cp Ti G4/CoCrMo		(ii) As the solution became more acidic, an increase in galvanic current values was observed.
	Ti UFG^1^ / CoCrMo		
Barros, camila 2020 [21] in vitro	Ti6Al4V/NiCr	(i) 0.9% NaCl at 227 ppm of F-, 2270 ppm F− and 12300 ppm F−	(i) The corrosion resistance of Ti6Al4V decreases with increasing fluoride concentration.
		(ii) pH 5, 5 and 4, 0	(ii) This decrease in resistance is more important in an acidic
		(iii) OCP	(iii) Environment fluorinated except ppm 227 of F− at pH 5.5.
		(iv) 15 days	
		(v) 1	
Mellado-valero et al. 2018 [2] in vitro	Ti G2/Au	(i) ASF pH 6, 5 and ASF pH3	(i) In the ASF pH 6.5.
	Ti G2/NiCrTi		(ii) The galvanic corrosion resistance has decreased compared to the results obtained in AS.
	Ti G2/CoCr	(ii) OCP, CP^5^ and ZRA.	(iii) In the ASF pH 3.
	Ti G2/CoCr-c	(iii) 30 min for OCP and 4 hours CP	(iv) The NiCr/Ti alloy loses its passivity and actively dissolves.
	Ti G2/Ti6Al4V	(iv) 0.28: CoCr-c and NiCrTi	(v) The TiG2/Ti6Al4V couple shows a huge increase in corrosion rates.
		(v) 5: CoCr, Ti6Al4V, AuPd	(vi) The Au alloy showed the most noble electrochemical behavior among all the materials studied.
Anwar, E.M. et al. 2011 [11] in vitro	Cp Ti/CM^6^ (NiCr)	(i) AS pH of 7.5	The addition of fluoride caused a significant decrease in the corrosion resistance of various couples, mainly those of Ti6Al4V.
	Cp Ti/CC^7^	(ii) NaF added to AS: three different concentrations were tested:	
	Ti 6Al4V/CM (NiCr)	(iii) 0, 01 M, M 0, 05 and 0.1 M	
	Ti6Al4V/CC	(iv) OCP and EIS^8^	

^1^Ti UFG: cold worked nanostructured cp Ti G4;^2^ F: fluoride; ^3^OCP: open circuit potential; ^4^ZRA: zero-resistance-ammeter; ^5^PC: potentiodynamic curves; ^6^CM: ceramic-metal; ^7^CC: all-ceramic; ^8^EIS : Electrochemical Impedance Spectroscopy.

**Table 3:** The evaluation of the oxidation surface.

Study	Galvanic couples	Environment, pH, period, method, area ratio, etc.	Results
Barros, camila 2020 [21] in vitro	Ti6Al4V/NiCr	(i) 0.9% NaCl at 227 ppm of F−, 2270 ppm of F−	A significant increase in roughness with increasing fluoride concentration and decreasing pH; the surface of Ti6Al4V coupled with NiCr of 12300 ppm F− solution at pH 4.0 showed an increase in roughness compared to that of 227 ppm F− solution at pH 5.5.
		(ii) 12300 ppm of F−, pH 5,5 and 4,0	
		(iii) Confocal microscope	
		(iv) 15 days	
		(v) 1	
Tuna et al. 2009 [12] in vitro	Ti cp (G 4)/Pd	(i) AS, pH 6.7, at 37°C	Significant fractures were observed by SEM on the surface of the superstructures of the Ti cpG4/NiCr and Ti cp/CoCr pairs. However, there were still few unaffected areas appearing weakly attached to the surface that could be detected. When the surfaces of the Ti cp/Pd and Au materials were studied, almost no visible effect has been revealed.
	Ti cp (G 4)/Au	(ii) Scanning electron microscopy (SEM)	
	Ti cp (G 4)/NiCr	(iii) 14 H	
	Ti cp (G 4)/CoCr	(iv) 0.33	
Oh and Kim 2004 [14] in vitro	Ti cp (G3)/gold	(i) AS at 37°C	Black spots were observed by SEM on the surface of the titanium connectors in all pairs.
	Ti cp (G 3)/NiCr	(ii) Optical microscope.	
	Ti cp (G 3)/CoCr	(iii) 5000s	
	Ti cp (G 3)/Ti cp (G 3)		

**Table 4:** The evaluation of metal ion release.

Study	Galvanic couples	Environment, pH, period, method, area ratio, etc.	Results
Barros and Camila 2020 [21] in vitro	Ti6Al4V/NiCr	(i) 0.9% NaCl at 227 ppm of F−, 2270 ppm of F, and 12300 ppm of F−, pH 5,5 and 4,0	A concentration of released Ti ions of 174.05 ppm in 12300 ppm of F− at pH 4.0 and 0.059 ppm in 227 ppm of F− at pH 5.5.
		(ii) ICP-MS^1^	Quantification for V ions gave 0,54 ppm in 12300 ppm of F− at pH 4.0 and 0,028 ppm in 227 ppm of F− at pH 5.5.
		(iii) 15 days	
		(iv) 1	
Bortagaray et al. 2016 [8] in vitro	Ti cp/noble alloys	(i) AS pH 7,1	Noble alloys with high gold and palladium content combined with cp titanium implants showed minimal release of metal ions into the environment.
	Ti cp/Ti cp	(ii) Analytical technique by static immersion.	
		(iii) 3 months	
Lee JJ. et al. 2015 [22] in vitro	Ti cp/Ni–Cr–Be	(i) DMEM^2^ + des	Release of metal ions was enhanced by galvanic corrosion due to contact between the base metal and titanium.
	Ti cp/NiCr	(ii) L-929 mouse	The amount of metal ions released and the cytotoxicity of the Ni–Cr alloy with beryllium was greater than that of other Ni–Cr alloys not containing beryllium.
	Ti cp/Ni-high Cr	(iii) Fibroblast cells.	
		(iv) ICP-MS,	
		(v) 48 hours	
Yamazoe M. 2010 [23] in vitro	Ti cp et Ti6Al4V/Ti cp, Ti6Al4V	(i) Lactic acid at 1% at 37°C	The level of Ti ion release was influenced by the microstructure of titanium. It was lower when the grain size was smaller. In the titanium-titanium combinations, the differences in the microstructure of the metal also markedly influenced the ionic release.
	Ti/noble alloys	(ii) ICPE^3^, SCLM^4^	
	With different surface roughness	(iii) 3 months	
Tuna et al. 2009 [12] in vitro	Cp Ti (G4)/Pd	(i) SA, pH 6.7, at 37°C	Higher total ionic concentration was observed in nonprecious alloys while precious alloys and titanium had much lower ionic concentration.
	Cp Ti (G4)/Au	(ii) ICP-MS,	
	Cp Ti (G4)/NiCr	(iii) 14 H	
	Cp Ti (G4)/CoCr	(iv) 0.33	
Foti et al. 1999 [24] in vivo	Cp Ti/precious alloy	(i) 8 implants Ti cp in the mandible of three primates	(i) After 2 months.
	Cp Ti/Ti	(ii) Histological analysis	(ii) Absence of titanium ions on the 48 regions explored.
		(iii) 2 months	(iii) The sectors with titanium superstructures.
			(iv) Migration of titanium to the area around the cervical region of the implant occurred in the presence of a precious alloy. This phenomenon did not occur with a titanium superstructure.

^1^ICP-MS: inductively coupled plasma-mass spectrometry; ^2^DMEM : Dulbecco's Modified Eagle Medium; ^3^ICPE: inductively coupled plasma; ^4^SCLM : atomic emission spectrometry.

## Data Availability

All data used in this review are available on Pubmed, Google Scholar, Scopus, ScienceDirect, EbscoHost, and Web of Science.
